# Avoidance and Endurance Responses to Pain Before and with Advanced Chronification: Preliminary Results from a Questionnaire Survey in Adult Patients with Non-Cancer Pain Conditions

**DOI:** 10.2147/JPR.S464509

**Published:** 2024-07-25

**Authors:** Karolin Teichmüller, Andrea Kübler, Heike L Rittner, Gudrun-Karin Kindl

**Affiliations:** 1 University Hospital Würzburg, Department of Anaesthesiology, Intensive Care, Emergency and Pain Medicine, Centre for Interdisciplinary Pain Medicine, Würzburg, Germany; 2 University of Würzburg, Institute of Psychology, Department of Psychology I, Würzburg, Germany

**Keywords:** Avoidance-Endurance Model, fear-avoidance, chronic pain, chronification, PAIN2020

## Abstract

**Purpose:**

The Avoidance-Endurance Model postulates fear-avoidance responses and endurance responses as important psychological mechanisms in the development and maintenance of chronic pain. The present study aims to investigate potential differences in avoidance and endurance responses to pain before and with advanced chronification.

**Patients and Methods:**

Two samples of adults with non-cancer pain at two different stages of chronicity were compared: One with pain and risk factors for chronicity (n=26, part of the PAIN2020 project) and one with chronic pain (n=33 from a pain day care clinic). The German Pain Questionnaire, the Graded Chronic Pain Scale (GCPS) and medical reports were used to measure duration and severity of pain. Responses to pain were assessed with the Avoidance-Endurance Questionnaire (AEQ) and psychological strain with the Depression, Anxiety and Stress Scales (DASS).

**Results:**

Both groups were primarily affected by musculoskeletal pain. Although not yet chronified, the risk group reported comparable GCPS levels of pain intensity and disability. Depression and stress ratings were also similar, except for anxiety, which was significantly elevated in the chronic pain sample (*p*<.001). The AEQ scales did not differ between groups, neither on any of the fear-avoidance- nor endurance-related dimensions. A post-hoc regression analysis revealed a significant prediction of fear-avoidance by pain-related disability (*p*<.001). The regression model for endurance responses was not significant.

**Conclusion:**

Patients with risk factors of chronification experience substantial pain-related burden. Responses to pain in the realm of the Avoidance-Endurance model do not appear to vary as a function of chronification. While fear-avoidance and pain-related disability correlate positively, endurance could not be associated to any of our variables.

## Introduction

The Avoidance-Endurance Model of pain^1^ describes different ways of responding to painful symptoms on a cognitive, emotional and behavioural level. The adaptive response pattern is characterised by a flexible, pragmatic coping style. The maladaptive patterns are labelled fear-avoidance pain responses (FAR) and endurance responses (ER). FAR is characterized by catastrophizing thoughts about pain and pain-related fear. FAR patients tend to avoid or interrupt potentially painful situations or activities, which increases disability. In contrast, ER comprises cognitive patterns of thought suppression and distraction, which allow ER patients to continue activities despite experiencing pain.^2^ ER can be further differentiated into eustress endurance response (EER), including cognitions of ignoring pain, positive mood despite pain and persistence behaviour and distress endurance response (DER) characterized by thought suppression, anxious/depressive mood and persistence behaviour.^3^ The maladaptive patterns foster chronicity on different pathways: FAR by physical deconditioning and underuse, ER by extensive strain and physical overload.^4^ In patients with low back pain, FAR has been found to be associated with higher pain intensity, disability and fatigue compared to adaptive coping. Patients with a tendency to ER exhibited high physical load and constrained postures accompanied by high levels of pain.^5^

The impact of FAR and ER on the development and maintenance of musculoskeletal pain was confirmed in several studies, for example^2^,^6^ Furthermore, a systemic review and meta-analysis showed that also in mixed samples of chronic pain patients, both avoidance and overactivity are associated with poorer patient outcomes.^7^ However, there is a paucity of evidence regarding potential changes in FAR or ER during the course of chronicity.^3^ The Avoidance-Endurance Model posits that FAR and ER patterns are relatively time stable. Accordingly, Ruscheweyh et al,^8^ who investigated the role of avoidance and endurance in patients with migraine, found that patients’ FAR or ER patterns remained unchanged from before to after treatment. Hasenbring and Verbunt^3^ acknowledged that only “well-directed behavioural interventions” might modify individual FAR or ER patterns. Conversely, the authors posit that ER patterns might shift towards FAR in the presence of long-term pain, suggesting a potential variability in the course of a chronic pain condition. Mediation analyses support a pathway in which catastrophizing leads to avoidance in patients with post-concussion symptoms^9^ and a link between catastrophizing and pain that is mediated by avoidance in women with chronic vulvovaginal pain.^10^ In both studies, the impact of endurance in the associations of symptoms, thoughts and coping behaviour was less significant.

The aim of the present study was to explore FAR and ER reactions to pain in two different patient populations with various types of non-cancer pain: One sample of adult patients with pain at the beginning of a possible chronification process, and one sample in whom chronification had already advanced. We hypothesized that changes in the use of FAR and ER coping strategies would occur with increasing duration of pain and stage of chronicity. As previous studies have suggested that FAR is more crucial,^9^,^10^ it was hypothesized that lower ER and higher FAR would be observed in patients with longer experience of pain.

## Materials and Methods

We investigated differences in FAR and ER in two samples of adult patients. One group comprised patients with pain and risk factors for chronicity at the beginning of a possible chronification process (“risk group”). This group either suffered from pain of short duration (< 6 weeks) or pain that occurred in distinct episodes with significant pain-free intervals. The other group included patients who were already suffering from chronic pain conditions (“chronic group”). For both groups, inclusion was not limited to specific types of pain. The study was approved by the Ethical Review Board of University of Würzburg (225/18_z) and complies with the Declaration of Helsinki. Participants did not receive any compensation.

### Samples

The “risk group” was part of the PAIN2020 project, a multicentre study that was funded by the German Federal Ministry of Health Care (Innovationsfonds 01NVF17049). The PAIN2020 project started in April 2018 with a term of 48 months. Its objective was to enhance the delivery of healthcare to adult patients with pain and risk factors for chronicity in a randomized clinical trial.^11^ According to the study protocol,^11^ inclusion criteria were as follows: (1) age > 18 years, (2) persisting pain for at least 6 weeks or recurrent episodes of pain in the last 2 years, (3) pain-related limitations in daily life, and (4) risk factors for chronification according to the German clinical practice guideline for non-specific back pain.^12^ These include inability to work for at least 4 weeks, pain-enhancing cognitive and behavioural mechanisms, affective symptoms of depression or anger, and psychosocial strain.^13^ Patients with a preexisting diagnosis of chronic pain, strong opioid intake or an interdisciplinary multimodal pain therapy in the past 2 years were excluded. Other exclusion criteria were severe somatic or psychiatric conditions, cognitive impairment, language barrier and pending pension claims.^11^ In- and exclusion criteria were verified by a pain specialist during a clinical assessment prior to patient assignment to the PAIN2020 project.

The “chronic group” was recruited from the routine pain day care out-patient clinic at the Department of Anaesthesiology, Intensive Care, Emergency and Pain Medicine, Centre for Interdisciplinary Pain Medicine, at the University Hospital Würzburg. All patients in this group reported pain for at least 3 months and had been diagnosed with a chronic pain condition in an interdisciplinary multimodal assessment prior to the commencement of an interdisciplinary multimodal pain therapy. For the chronic group, exclusion criteria were limited to cognitive impairment and language barrier.

### Study Material and Procedure

The Avoidance-Endurance-Questionnaire (AEQ) was developed and validated by Hasenbring et al.^14^ Based on the Avoidance-Endurance Model, the AEQ assesses emotional, cognitive and behavioural responses to pain, which represent either FAR or ER. Through principal components analysis (PCA), Hasenbring et al^14^ identified five FAR-scales (*Anxiety/depression, Help-/hopelessness, Catastrophizing thoughts, Avoidance of social activities*, and *Avoidance of physical activities*) and four ER-subscales (*Positive mood despite pain, Thought suppression, Humor/distraction*, and *Pain persistence*). Participants indicate their feelings and thoughts in response to pain during the past 14 days on a scale from 0 (never) to 6 (every time). The AEQ has been applied in a variety of pain populations, including those with chronic back and neck pain,^15^ subacute low back pain,^6^ migraine,^8^ and experimentally induced pain.^16^ In the present study, internal consistency was found to be good to excellent for all AEQ scales (Cronbach’s α of .80 to .94), except for the Thought suppression scale, which exhibited acceptable reliability (α=.70).

The following demographic and clinical data were derived from the medical reports of each patient: Age, sex (male/female), pain-related and where applicable, psychiatric ICD-10 diagnoses, and the Mainz Pain Staging System (MPSS),^17^ a validated clinical instrument for measuring the development of chronic pain in three stages (I–III).

The German Pain Questionnaire^18^ was used to assess the duration of pain symptoms by a single-item question with six answering options on an ordinal scale: “less than 1 month”, “1 month – ½ year”, “½ year – 1 year”, “1–2 years”, “2–5 years”, and “more than 5 years”. Additionally, the German Pain Questionnaire incorporates the Graded Chronic Pain Scale (GCPS),^19^ a widely employed instrument for measuring pain intensity and pain-related disability. The GCPS distinguishes between four levels of pain severity: Grade 1: low disability-low intensity; Grade 2: low disability-high intensity; Grade 3: high disability-moderately limiting; Grade 4: high disability-severely limiting. The German version of the GCPS has been demonstrated to be reliable and valid in patients with back pain.^20^ Furthermore, the Depression, Anxiety and Stress Scales (DASS),^21^ a 21-item self-administered questionnaire, was employed, in which patients rate symptoms of depression, anxiety and stress on a 4-point scale. Higher scores indicate higher levels of the negative emotional states. The German version applied in our study has been validated by Nilges and Essau^22^ and demonstrated acceptable to excellent internal consistency in the present study (Anxiety: α= .72, Depression: α= .93, Stress: α= .83).

The participants in the risk group completed the German Pain Questionnaire, the AEQ and other study materials (not reported here) after providing written informed consent at the time of their inclusion in the PAIN2020 project (June 2019-July 2021).

The chronic group had completed the German Pain Questionnaire at the time of their admission to the day clinic as part of the clinical routine. They provided written consent to participate in the study and completed the AEQ during a regular group therapy session in December 2021 or January 2022.

Both groups underwent an interdisciplinary multimodal assessment as defined by Casser et al.^23^

### Data Analysis

Statistical analyses were conducted using IBM SPSS software (version 29). A statistical level of α ≤ .05 was considered significant. Group differences between the risk and chronic group were analyzed using unpaired *t*-tests for metric variables (age, DASS, AEQ). Shapiro Wilk tests for normality and Levene tests for homogeneity of variances were performed. For the AEQ scales of *Anxiety/depression, Avoidance of social activities, Avoidance of physical activities, Positive mood despite pain*, and *Thought suppression*, both normality and homogeneity were given. In all other cases, adjusted df and *t*-values (Welch test) were used for interpretation. Mann–Whitney *U*-tests were conducted for ordinal data (pain duration, MPSS, GCPS), and the Chi-square test for categorical variables (sex).

As it became apparent that the risk and chronic group did not differ in age, sex ratio, and GCPS scores (see below), we proceeded to investigate whether these parameters were associated with differences in FAR or ER. We calculated FAR and ER mean scores of the FAR and ER items and conducted two post-hoc multiple linear regressions to predict FAR and ER from age, sex, and pain intensity and pain-related disability of the GCPS. For both models, the assumptions of linearity, absence of outliners (± 3 *SD*), homoscedasticity, lack of multicollinearity, and normal distribution of residuals were met.

## Results

### Pain-Related and Psychological Diagnoses

Both groups were primarily affected by musculoskeletal pain. Non-specific back pain was the most prevalent diagnosis in the risk group, affecting 76.9% of patients. This was followed by joint pain, and muscle and tendon pain. Only two patients (7.7%) in the risk group fulfilled the criteria for a psychological ICD-10 diagnosis, namely *Chronic pain disorder with somatic and psychological factors (F45.41)*. These diagnoses were a result of the interdisciplinary multimodal assessment after inclusion into the PAIN2020 project and, according to the study protocol,^11^ did not result in a retroactive exclusion. In the chronic group, back pain, soft tissue and joint pain were the most frequent pain disorders. In addition, the chronic group exhibited a greater prevalence of miscellaneous pain-related diagnoses, as well as an accumulation of pain-related diagnoses and a markedly higher prevalence of mental comorbidities (Table 1).

**Table 1:** Pain-Related and Psychological Diagnoses

	ICD-10	Risk Group (n=26)	Chronic Group (n=33)
**Pain-related diagnoses** n (%)^a^			
Specific back pain	M47; M48; M50; M53	4 (15.4%)	8 (24.2%)
Non-specific back pain	M54	20 (76.9%)	21 (63.6%)
Joint pain	M05; M15; M17; M25	8 (30.8%)	18 (54.5%)
Muscle & tendon pain	M76; M79; M99; G71	7 (26.9%)	19 (57.6%)
Primary headache	G43; G44; G50	5 (19.2%)	11 (33.3%)
Neuropathies	G57; G62; G90	0	7 (21.2%)
Temporomandibular joint disorders	K07.6	0	2 (6.1%)
Unclassified pain in certain regions	R07; R10; R20	0	4 (12.1%)
**Number of pain conditions**			
1		12 (46%)	4 (12%)
2		10 (39%)	12 (37%)
3		4 (15%)	10 (30%)
4		0	4 (12%)
5 or more^b^		0	3 (9%)
**Psychological diagnoses** n (%)^a^			
Chronic pain disorder with somatic and psychological factors	F45.41	2 (7.7%)^c^	33 (100%)
Depression	F32; F33	0	19 (57.6%)
Neurotic & stress-related disorders	F40; F41; F42; F43	0	13 (39.4%)
Addiction	F17	0	1 (3.0%)
Personality disorders	F60	0	1 (3.0%)
Factors influencing health status and contact with health services	Z73	0	1 (3.0%)

**Notes**: ^a^% represent percentages of n=26 for the risk group and n=33 for the chronic group, respectively. Percentages do not add up to 100 because of multiple diagnoses per patient. ^b^The maximum number of pain-related diagnoses in the chronic group was 7 in n=1 patient. ^c^Before inclusion, all patients in the risk group had been asked if, to their knowledge, they had been diagnosed with chronic pain before. All denied. The assignment of the diagnosis F45.41 was a result of the interdisciplinary multimodal assessment after inclusion into the PAIN2020 project. PAIN2020 did not foresee a retroactive exclusion in these cases.

### Pain and Clinical Characteristics

Table 2 provides descriptive measures and the results of statistical group comparisons for all study variables.

**Table 2:** Descriptive Measures and Group Comparisons

	Risk Group (n=26)	Chronic Group (n=33)	Group Comparison
**Age** [years] *M ± SD*	48.85 (12.17)	46.00 (15.24)	*t*(56.98) = 0.798, *ns*.
**Male: female** n (%)	9 (34.6): 17 (65.4)	11 (33.3): 22 (66.7)	*Χ*^2^(1) = 0.011, *ns*.
**Pain duration** n (%)			*U*=237.00, *p*=.003
1 month – ½ year	7 (26.9)	1 (3.0)	
½ year – 1 year	6 (23.1)	4 (12.1)	
1–2 years	6 (23.1)	7 (21.2)	
2–5 years	3 (11.5)	11 (33.3)	
More than 5 years	4 (15.4)	10 (30.3)	
**MPSS** n (%)			*U*=169.50, *p*<.001
Stage I	9 (34.6)	–	
Stage II	14 (53.8)	15 (45.5)	
Stage III	3 (11.5)	13 (39.4)	
Missing	–	5 (15.2)	
**GCPS** n (%)			*U*=398.50, *ns.*
Grade 1	3 (11.5)	7 (21.2)	
Grade 2	4 (15.4)	5 (15.2)	
Grade 3	7 (26.9)	6 (18.2)	
Grade 4	12 (46.2)	15 (45.5)	
**DASS** *M ± SD*			
Depression	5.42 (2.50)	5.55 (5.07)	*t*(48.86)=−0.121, *ns*.
Anxiety	2.31(2.20)	5.55 (3.84)	*t*(52.59)=−4.066, *p*<.001
Stress	7.46 (2.85)	8.39 (4.39)	*t*(55.14)=−0.985, *ns*.

**Figure 1:**
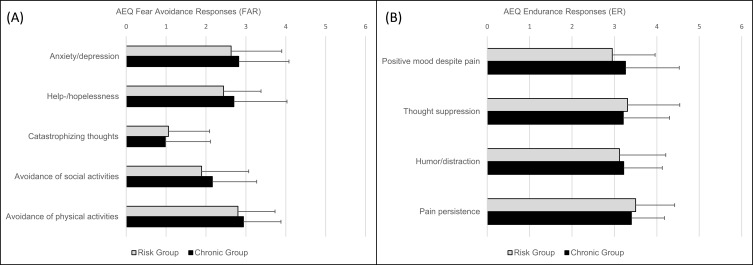
No differences in FAR and ER between the risk and chronic group.

**Notes**: Comparable means (±*SD*) of the AEQ FAR and ER subscales in the two study groups. Unpaired *t*-tests showed no significant difference on any of the FAR subscales ((**A**), −0.90<*t*_(57)_>0.30, all ns.) nor ER subscales ((**B**), −1.03<*t*_(57)_>0.47, all ns.) between the risk and chronic group.

The two study groups did not differ in age and sex ratio, but regarding pain duration and stage of chronicity (MPSS). As expected, the risk group reported significantly shorter duration of pain symptoms and held lower MPSS stages. The GCPS did not significantly differ between the groups, indicating that pain intensity and pain disability were comparably high in both groups. Of the DASS scales, only anxiety demonstrated a significant difference with higher scores observed in the chronic group. Depression and stress ratings were not significantly different. Both groups exhibited comparable levels of FAR and ER with no significant difference on any of the AEQ scales (Figure 1).

### Post-Hoc Analyses

Since the risk and chronic group did not differ in age, sex ratio and GCPS scores, we explored if these parameters were associated with differences in FAR or ER by multiple linear regressions (Table 3).

**Table 3:** Multiple Linear Regressions for FAR and ER

Coefficients	FAR		ER	
**Beta**	**t**	**Beta**	**t**
Intercept		4.168***		6.266***
Age	.009	0.071	−.015	−0.111
Sex	−.123	−1.057	.100	0.742
Pain intensity	−.226	−1.551	.092	0.551
Pain-related disability	.609	4.361***	−.237	−1.475
**Model summary**	*F*(4, 54) = 5.32***		*F*(4, 54) = 0.72, *ns*.	
**Goodness of fit**	*R*² = .28, *R*²_Adjusted_ = .23		*R*² = .05, *R*²_Adjusted_ = −.02	

**Note**: ***p ≤ .001.

For FAR, the goodness-of-fit according to Cohen^24^ for the overall model was medium to high. The overall regression was statistically significant, with pain-related disability identified as the sole significant predictor of FAR. Conversely, ER could not be predicted from any of the variables.

## Discussion

In contrast to our expectations, pain duration, stage of chronicity and pain intensity were not associated with FAR or ER responses to pain suggesting a trait nature of FAR and ER. However, it should be noted that our study was a between-design comparison of two samples, which limits the ability to draw conclusions about intraindividual changes of FAR or ER over time. A prospective longitudinal study design would be necessary to address this limitation.

Patients with pain and risk factors for chronicity presented with levels of pain intensity and pain-related disability that were comparable to those of patients with chronic pain conditions. In line with this, Hüllemann at al. found no differences in average pain intensity between large numbers of patients with subacute vs chronic low back pain and even higher ratings of maximum pain in the subacute sample.^25^ In contrast our findings, however, Hüllemann et al observed an increase in disability from the subacute to the chronic stage.^25^ A smaller study comparing patients with subacute vs chronic back pain regarding depression, found no differences between the two groups, but elevated symptoms in both groups compared to healthy controls.^26^ In accordance with our findings of comparable levels of depression and stress, this indicates a considerable psychological burden already at the beginning of a possible chronification process. This corroborates the strong connection and mutual development of pain and negative affect.^27^ Consequently, we posit that the necessity for professional, pain-related healthcare in both groups must be regarded as equally high. Indeed, experts concur that multidisciplinary assessment and multimodal pain therapy should not only be provided for patients with chronic pain symptoms, but also for those in an early state of the disease (see^12^ for non-specific low back pain and^23^,^28^ for other types of pain). This view is supported by our data.

Anxiety, as measured by the DASS, was the only parameter in our study that exhibited a significantly higher level in the chronic group as compared to the risk group. This suggests that anxiety increases during the process of chronification, although there are conflicting results using a different measure of anxiety.^25^ When pain persists, concerns about financial and occupational issues may arise as well as conflicts in the patient’s social relationships. This may manifest in mental comorbidities, which could explain the high prevalence of depressive and anxiety disorders observed in our chronic group. Nevertheless, the elevated level of anxiety (measured by the DASS) in the chronic group did not result in higher FAR on the AEQ.

The role of fear-avoidance in the development and maintenance of pain conditions is emphasized in the fear-avoidance model of pain by Vlaeyen et al.^29^ There is a long-lasting history of theoretical and empirical evidence for the importance of pain-related fear in chronic musculoskeletal pain.^30^,^31^ Also for chronic post-surgical pain, a systematic review and meta-analysis by Giusti et al^32^ has recently confirmed that anxiety was the main psychological risk factor. Moreover, fear and distress appear to represent risk factors for poor treatment outcome.^6^,^33^ In contrast, endurance was unrelated to any of our study parameter. Nevertheless, previous studies have shown that ER is frequent and clinically relevant,^6^ which is why we consider further research and concretization desirable. Further clarification is required on a conceptual and empirical level regarding the impact of ER on the process of chronification, particularly in relation to the possible pathways between endurance and pain perception as well as disability.^34^

Due to the limited size of our sample, the current analysis lacks the statistical power to detect group differences of small effect size. Consequently, the conclusions drawn must be considered preliminary. Subgroup analysis, for example, of gender differences or disease-specific patterns of FAR and ER would be of interest, especially given that the sample includes various types of non-cancer pain conditions. Unfortunately, the limited number of participants precludes the possibility of such calculations. Furthermore, the present results are based solely on self-reported data. The inclusion of additional sources of information, such as behavioural observation, proxy-ratings of FAR and ER or objective parameters of physical functioning would be beneficial. Furthermore, it should be acknowledged that the chronification of pain is driven by numerous other variables that were not part of the present study, such as occupational or iatrogenic factors.^12^ Individual-level occupational factors, so-called “blue flags”, include, amongst others, physical job demands, workplace social support or dysfunction, and fear of re-injury.^35^ A systematic review on health care professional beliefs and attitudes found that physicians and other paramedical therapists with elevated fear avoidance beliefs are more likely to advise patients to reduce work and physical activities.^36^ It seems likely that this, in turn, promotes fear-avoidance beliefs in the patients.

## Conclusion

The findings of this study indicate that patients with pain and risk factors of chronicity experience a significant pain-related burden already at the beginning of a possible chronification process. However, their responses to pain in the realm of the Avoidance-Endurance Model do not appear to differ from those of patients in whom chronification has already taken place. While endurance could not be associated with any of the variables in this study, fear-avoidance and pain-related disability correlated positively. Consequently, we consider anxiety and fear-avoidance behaviour to be promising targets for interdisciplinary multimodal intervention in the early stages of a pain disease as well as in patients with chronic conditions.  

## Abbreviations

AEQ, Avoidance-Endurance Questionnaire; DASS, Depression, Anxiety and Stress Scales; DER distress endurance responses to pain; ER, endurance responses to pain; EER, eustress endurance responses to pain; FAR, fear-avoidance responses to pain; GCPS, Graded Chronic Pain Scale; ICD-10, International Statistical Classification of Diseases and Related Health Problems 10th version; MPSS, Mainz Pain Staging System.
